# Cell Adhesion Molecules in Neuroblastoma: Complex Roles, Therapeutic Potential

**DOI:** 10.3389/fonc.2022.782186

**Published:** 2022-04-27

**Authors:** Briana E. Heinly, Christa N. Grant

**Affiliations:** ^1^ Penn State College of Medicine, Hershey, PA, United States; ^2^ Division of Pediatric Surgery, Milton S. Hershey Medical Center, Hershey, PA, United States

**Keywords:** neuroblastoma, cell adhesion molecules, tumorigenesis, metastasis, integrin, cadherin, NCAM

## Abstract

Neuroblastoma, a biologically heterogeneous tumor derived from neural crest cells, accounts for approximately 15% of childhood deaths from cancer. Recently, scientific literature has explored the role of cell adhesion molecules (CAMs) in cancer metastasis through cell detachment, migration, and invasion. Through a review of the current literature, it is evident that expression of different CAMs on neuroblastoma tumors is associated with favorable or unfavorable clinical prognosis. In patients diagnosed with neuroblastoma, treatment strategies include chemotherapy, surgery, radiotherapy, stem cell transplant, and more recently, immunotherapy and other targeted therapies. Long term survival remains poor despite multimodality treatment, especially for children with high-risk neuroblastoma, making it more necessary to explore innovative targeted therapies. CAMs have immense potential as therapeutic targets, but there is a need for growth and scientific exploration before CAM therapies become clinically useful.

## Introduction

In healthy tissues, cell adhesion molecules (CAMs) promote homeostasis between cells and between cells and the extracellular matrix. There are four main groups of CAMs: cadherins, integrins, selectins, and immunoglobulins (1). CAMs are primarily glycoproteins expressed on the cell surface membranes, and work by binding via their extracellular domains (1, 2). Recently, CAMs have been investigated more for their role in cancer and metastasis. Given that they are important in the interactions between other cells and the extracellular matrix, they also regulate motility, migration, and metastasis in cancer cells (1, 2). The overexpression or under-expression of CAMs can greatly affect the metastatic potential and progression of disease.

Neuroblastoma is a tumor derived from the precursor cells of the sympathoadrenal lineage during development (3). Neuroblastoma accounts for approximately 15% of childhood deaths from cancer; it is the most common extracranial solid tumor in children and is often metastatic at the time of diagnosis (2). Neuroblastomas are known to be biologically heterogeneous, and these differences result in different clinical outcomes for patients (4); overall, outcomes remain bleak for high-risk tumors, with long term survival around 50%. The categorization of neuroblastomas is determined by the amount of cellular differentiation in the tumor. Well-differentiated tumors, with abundant ganglion cells, are called ganglioneuromas, and they have a better prognosis than neuroblastomas, which have abundant neuroblast cells and a poor prognosis (5).

In recent literature, there have been many studies conducted to determine what oncogenic factors contribute to neuroblastoma formation. Hyperdiploidy, genetic aberrations, and v-myc myelocytomatosis viral related oncogene (MYCN) amplification are among the most widely accepted genomic and genetic causes of neuroblastoma (3). The gain of 17q, loss of 1p or 11q, anaplastic lymphoma kinase (ALK) mutations, and MYCN amplification all correlate with poor prognosis, while hyperdiploidy is associated with improved prognosis (3, 5).

Currently, the treatment of neuroblastoma includes multi-modal therapy using chemotherapy, surgery, radiotherapy, and stem cell transplantation (6). Immunotherapy and targeted therapies such as anti-GD2 monoclonal antibody Dinutuximab have added immense value as second line agents in recent years (7, 8). These treatment methods are not without risk, and can lead to damage to internal organs, immune compromise, severe infections, mucositis, secondary malignant neoplasm, anemia, infertility, and hair loss (7). Aside from the obvious side effects, there is also limited use for these therapies because of neuroblastoma’s biological and immunological features, which means that there is an increased need for novel targeted therapies that could be more effective.

In this review, we focus mainly on the effects of CAMs in neuroblastoma, seeking to identify potential avenues for therapeutic advantage. The CAMs that have been the most widely studied in neuroblastoma include N-cadherin, E-cadherin, neural cell adhesion molecule (NCAM) and polysialylated-neural cell adhesion molecule (PSA-NCAM), as well as a variety of integrin subunits. Throughout this review, the involvement and effect of these cell adhesion molecules, along with activated leukocyte cell adhesion molecule (ALCAM) and intercellular adhesion molecule 2 (ICAM-2) and potential for therapeutic targets in neuroblastoma is discussed. The English language literature was searched for all publications related to neuroblastoma and cell adhesion molecules with no date limitation. Papers that showed a direct relationship between a cell adhesion molecule and neuroblastoma progression, tumorigenesis, or metastasis were included in the review. All manuscripts addressing secondary, tertiary, or further downstream effects/effectors were excluded. No human or animal subjects were involved in this research. All authors are up to date on research ethics training.

## Cadherins

Cadherins are a class of type-1 transmembrane proteins which depend on calcium (Ca2+) ions to mediate cell-cell adhesion. In regard to neuroblastoma, the most extensively studied cadherins are neural (N-) cadherin and epithelial (E-) cadherin, which are thought to play a significant role in the metastatic cascade (9). E-cadherin is found mainly on epithelial cells and functions to form tight cell-cell associations (9). N-cadherin is primarily found in neuronal tissues and fibroblasts, and its downregulation is crucial for migration of neural crest cells during embryonic development (9). Other cadherins of interest, such as vascular endothelial (VE-) and osteoblast (OB-) cadherin, are associated with blood vessel endothelium, and therefore are associated with tumor vasculature (10).

In malignancies, there is a well-known “cadherin switch”, that once flipped, promotes metastasis. The “cadherin switch” refers to the loss of E-cadherin mediated cell-cell adhesion and the concurrent *de novo* change in expression of N-cadherin, which promotes cell motility and migration (9). Cells undergoing this E-cadherin-mediated switch to metastasis lose their polarity, become invasive, and resist apoptosis (10).

## Cadherins in Neuroblastoma

### E-Cadherin

To this point, not much research has been done specifically on the role of E-cadherin in neuroblastoma tumors. E-cadherin is primarily found on epithelial cells, and therefore would not be expected to be expressed on neuroblastoma tissue. This was investigated by Gluer et al. (11), who looked at the expression of cell adhesion molecules and intermediate filaments in several childhood tumors (11). They found that E-cadherin was not expressed in either the neuroblasts or ganglion cells in their neuroblastoma samples.

### N-Cadherin

N-cadherin is mainly found in normal neuronal tissues and fibroblasts (9). In neuroblastoma cells, Reyes-Mugica et al. found that N-cadherin is localized to the cytoplasm as well as sites of cell-cell contact by performing immunofluorescence (12). They also confirmed that cell adhesion is mediated by N-cadherin, as knockdown of N-cadherin with a monoclonal antibody inhibited cell-cell adhesion (12). The control of N-cadherin expression allows correct migration of the neural crest cells during early embryonic development, and downregulation of N-cadherin allows for the migration of cells away from the neural tube (9). In similar fashion, downregulation of N-cadherin in tumor tissues allows for tumor cells to break away from the original tumor site and travel to distant tissues (9).

Lammens et al. used ten neuroblastoma cell lines to create 365 neuroblastoma tumor samples, and then correlated the tumor N-cadherin expression with the stage and risk stratification (9). They found that N-cadherin was expressed on all the neuroblastoma tumors, however, the level of expression correlated to very different clinical outcomes. The median N-cadherin mRNA expression level was significantly lower in metastasized neuroblastoma tumors (stage 4 and 4S) when compared to localized tumors (stages 1, 2, and 3) (p =0.006) (9). Patients with higher risk disease, which was categorized as patients with a stage 4 tumor at an age over 1 year or stage 1, 2, 3, or 4S tumors with MYCN amplification, also had lower N-cadherin mRNA expression compared to low-risk patients (p = 0.048). Low N-cadherin expression was also strongly associated with an unfavorable status *via* the International Neuroblastoma Pathology Classification (INPC) prognostic system (p =0.004) (9).

Reyes-Mugica et al. suggests that there is also a link between N-cadherin and the deleted in colon cancer (DCC) protein, which is commonly aberrant in neuroblastoma (12). Reyes-Mugica et al. overexpressed full-length and truncated DCC constructs in neuroblastoma cell lines and found that colonies produced by clones with truncated DCC had a scattered morphology, whereas full-length DCC transfected clones had a more epithelioid morphology (12). They found that this change was not mediated by the DCC protein itself, but rather the decrease of N-cadherin expression that follows the overexpression of truncated DCC. In contrast, full-length DCC transfected clones led to an increase in N-cadherin expression, which then led to stronger cell-cell adhesion and a primarily epithelioid morphology (12). This again suggests that a decrease in N-cadherin expression is associated with decreased cell-cell adhesion and a greater potential for tumor cell migration.

### Other Cadherins

Other less common cadherins have been shown to play a role in neuroblastoma progression. Placental (P-) cadherin, also known as placental cadherin, is overexpressed in unfavorable neuroblastomas (4). Hiyama et al. suggest that this overexpression of P-cadherin might be associated with the infiltrative capacity of tumor cells (4). Not all cadherins are tumor promoting; a study by Takeuchi et al. showed that truncated (T-) cadherin expressing neuroblastoma cells lost their mitogenic proliferative response to epidermal growth factor (EGF). Since EGF is required for the proliferation of neural stem cells, T-cadherin may function as a negative regulator of neural cell growth (13).

Other cadherins, such as VE-cadherin and OB-cadherin, play a more important role in the formation and maintenance of tumor vascular structures (10). VE-cadherin is present within the endothelial cells in blood vessels and provides stability in the junctions between endothelial cells (10). OB-cadherin expression is often aberrant alongside N-cadherin expression, which suggests that this could also be associated with metastatic potential (10). There have also been studies done in prostate cancer that suggest OB-cadherin could regulate the process of bone metastasis (14). Blaschuk et al. suggests that in this way, a cocktail of N-, OB- and VE-cadherin antagonists could be ideal for an anti-cancer therapy (10).

## NCAM and Neuroblastoma

Neural cell adhesion molecule (NCAM), also known as the CD56 antibody, is a homophilic glycoprotein that is expressed on the surface of neurons, glia, and skeletal muscle. It has been widely proven that NCAM is present on neuroblastoma cells, as they arise from neural lineage. NCAM binds to other cell adhesion molecules from different classes, such as cell adhesion molecule L1, fibroblast growth factors, and extracellular matrix (ECM) components (15). Some studies suggest that there is an inverse correlation between NCAM expression in neuroblastoma cells and tumor cell adhesiveness (16, 17). With higher levels of NCAM expression, cells have more intense homophilic tumor binding, and are only loosely adherent to heterophilic cells (16). With lower levels of NCAM expression, cells have higher heterophilic binding capacity, which promotes disaggregation from the original tumor site and migration to distant tissues (16). In tumors that have metastasized, there are often high serum levels of NCAM, and this could be due to the NCAM being released from the tumor cell surface into the serum.

There are three main forms of NCAM, including 120, 140, and 180 kDa NCAM, as well as numerous less-known splicing variants (18). Among the spectrum of neuroblastic tumors (neuroblastoma, ganglioneuroblastoma, and ganglioneuroma), the expression of the different NCAM variants depends on the degree of differentiation of cells. Ganglioneuroma and ganglioneuroblastoma show significantly higher NCAM 120 expression compared to neuroblastomas (p = 0.0007) (18). Neuroblastomas, on the other hand, have a much higher expression of the NCAM 180 isoform, whereas the NCAM 140 isoform is not statistically different between the three differentiation stages (18). Interestingly, NCAM 140 kDa is associated with neurite outgrowth, and NCAM 180 kDa is associated with cell motility (19). This could potentially explain the higher metastatic potential of neuroblastoma tumors compared to ganglioneuroblastoma and ganglioneuroma.

Two other types of NCAM, close homolog of L1 protein (CHL1) and neuronal glia related cell adhesion molecule (NrCAM) are also associated with tumorigenesis and progression in different malignancies (20). Wachowiak et al. studied the expression of these two CAMs in neuroblastoma specimens of 56 children and found that both CHL1 and NrCAM were detected in patients with low-grade neuroblastoma (20).

In addition to having different types and isoforms, NCAM can also be post-translationally modified by the addition of polysialylated acid (PSA). PSA is involved in a decrease in cell adhesion and therefore an increase in cell migration (21). Notably, PSA as only found to be expressed on isoforms NCAM-140 and NCAM-180, which are the two isoforms that are mainly expressed in the less differentiated/more malignant tumor types (17). In a study done by Valentiner et al., they grew 5 different neuroblastoma cell lines in mice (17). In their model, neuroblastoma cell lines that were strongly positive for PSA-NCAM had lower tumor take-rates than PSA-NCAM-negative cell lines. Cells positive for PSA-NCAM were able to disseminate and migrate as single cells into the lung parenchyma, promoting distant metastasis. Cell lines with little to no PSA-NCAM expression still metastasized, but they were only able to do so at a vascular level, suggesting that PSA-NCAM promotes both migration and invasion into distant tissues (17). This supports findings by Blaheta et al., which imply that NCAM must be downregulated or polysialylated before or during the initial contact with endothelial cells before adhesion and penetration can take place (16).

Seidenfaden et al. established that PSA controls tumor cell growth and differentiation by interfering with NCAM signaling at cell-cell contacts. Upon removal of PSA from the cell surface, there was reduced proliferation and increased cell differentiation *via* the activation of extracellular signal-regulated kinases (15). This activation of extracellular signal-regulated kinases and subsequent neuronal differentiation in neuroblastoma cells leads to a higher level of differentiation and a more favorable prognosis. Therefore, the addition of PSA onto NCAM to form a PSA-NCAM complex keeps the neuronal cells in an undifferentiated state, with high metastatic potential.

Given that PSA-NCAM is only expressed on mainly undifferentiated tumors, it can be helpful in the diagnosis of neuroblastoma and differentiation from other childhood neuronal tumors (22). Expression of PSA-NCAM correlates with tumor progression, metastasis, and invasion in several tumors (17, 23). Gluer et al. designed a study to evaluate whether PSA-NCAM was a helpful marker in distinguishing between various embryonal tumors. They found that PSA-NCAM was expressed on the surface of neuroblastoma, rhabdomyosarcoma, and Wilm’s tumor (11). Patients with Ewing sarcoma, primitive neuroectodermal tumor, and lymphoma were all negative for PSA-NCAM in both immunohistochemistry and serum testing (11). This could be helpful in distinguishing between these “small round blue cell” tumors of childhood, which is a known histopathologic difficulty. The study conducted by Gluer et al. provides promising information suggesting that PSA-NCAM could be helpful in the diagnosis of neuroblastoma, however the sample size was very limited (n=17), so the diagnostic potential of PSA-NCAM warrants further investigation (11).

The presence of PSA-NCAM is becoming recognized as an important prognostic factor for neuroblastomas. This is supported by Korja et al., who found that 17 out of 20 NCAM-positive primary neuroblastomas also expressed PSA-NCAM, and those that did express PSA-NCAM were also the most advanced/metastatic tumors at diagnosis (24). Gluer et al. also found similar results in their study of 27 ganglioneuroma and neuroblastoma tumor specimens: PSA-NCAM expression was highest in patients with undifferentiated neuroblastoma and advanced stages of disease, while differentiated tumors and low clinical stages had distinctly reduced to no PSA-NCAM expression (25). Six out of eleven ganglioneuroma and ganglioneuroblastoma species had no reactivity against PSA-NCAM, while higher-stage ganglioneuroma and some ganglioneuroblastoma samples had moderate to high reactivity (25). This further supports the claim that PSA-NCAM is expressed mainly in undifferentiated and higher-risk tumors. In a different study by Gluer et al., they found that serum levels of PSA-NCAM were also dramatically elevated (more than sixfold) in children with advanced neuroblastoma stages compared to healthy children (26). Serum levels ranging between 30.7 and 301.3 kU/L were found in children with neuroblastoma, as well as one extremely high case that had serum levels of 1377 kU/L. The mean serum level found in healthy children was 26.8 kU/L, which is significantly less than the values found for neuroblastoma patients. For reference, serum levels over 20 kU/L in adults are considered pathological, however levels are known to decrease with age (26).

Interestingly, there has been some conflicting information about whether PSA-NCAM expression correlates with MYCN amplification. Gluer et al. found that there was no correlation between PSA-NCAM and MYCN amplification, but Korja et al. found that PSA-NCAM was a strong unfavorable prognostic indicator for metastatic disease, especially in MYCN amplified tumors (24, 26). However, in their study looking at serum levels of PSA-NCAM, Gluer et al. found that MYCN amplification was found in the three children with the highest serum levels of PSA-NCAM, but amplification was not present in tumors of patients with moderately raised or normal serum PSA-NCAM levels (26). This suggests that the correlation between PSA-NCAM and MYCN may just not be noticeable until a more advanced disease stage. In comparison to the prognostic value of MYCN, one of the main prognostic indicators in neuroblastoma, negative PSA-NCAM and NCAM expression were even stronger predictors of unfavorable outcomes (24) (**Table 2**).

In addition to its value as a prognostic factor, PSA-NCAM may also be a good marker for disease progression during the treatment process. Gluer et al. followed 15 children during treatment and noted that serum PSA-NCAM concentrations decreased to normal within a few weeks of initiating chemotherapy treatment, or by a few days after complete surgical resection (25). The sample size limits the power of the study, so it would be worthwhile to investigate the use of PSA-NCAM for disease progression on a larger scale.

## ALCAM and Neuroblastoma

Activated leukocyte cell adhesion molecule (ALCAM) is a member of the ‘immunoglobulin superfamily’. It mediates homotypic and heterotypic interactions between tumor cells and the extracellular matrix. It has been suggested as a possible prognostic factor for tumor staging. Wachowiak et al. studied the effects of ALCAM on neuroblastoma and found that weak ALCAM was significantly correlated with positive MYCN expression as well as decreased recurrence-free and overall survival (27). Therefore, ALCAM could be a potential prognostic marker for neuroblastoma.

## ICAM-2 and Neuroblastoma

Intracellular adhesion molecule-2 (ICAM-2) is a transmembrane glycoprotein also belonging to the immunoglobulin superfamily. The interaction between ICAM and neuroblastoma was detailed by Feduska et al., who sought to evaluate the impact of ICAM-2 on neuroblastoma cells (28). They found striking results, showing that ICAM-2 was able to reverse the metastatic phenotype in neuroblastoma cells. However, this relationship was dependent on a critical intermediate, α-actinin. In the presence of α-actinin, ICAM-2 was able to suppress metastatic potential in preclinical models (28). Of note, ICAM-2 was not able to suppress the tumorigenic potential, but this effect on metastasis could have promising clinical uses.

## Integrins and Neuroblastoma

Integrins are another family of cell adhesion molecules that are responsible for adhesion mainly to the extracellular matrix. Different subunits, including many versions of alpha and beta subunits, can be produced and combine to form an integrin receptor. Depending on the combination of integrin subunits, there can be wildly different effects of the receptor on cell function. In neuroblastoma, some integrins are associated with a better prognosis, while others are associated with a worse prognosis.

Integrin subunits found to have a favorable prognosis include α1, α2, α4, α6, αν, β3 and β4 (29, 30). Tunaka et al. found that neuroblastoma cell lines that expressed α1 integrin also had less MYCN amplification. When MYCN was overexpressed in these cell lines, they saw that α1 integrin was consequently downregulated. This inverse relationship between α1 integrin and MYCN confirms that it is a favorable CAM in neuroblastoma (30). Favrot et al. was the first to comprehensively evaluate integrin expression on neuroblastoma tumor specimens (29). Their study analyzed the integrin expression on 45 neuroblastoma specimens from a variety of histological and clinical stages (29). They found that the α2 and α6 integrin subunits were associated with a more favorable prognosis, as they were mainly expressed on low-grade, well-differentiated specimens. The specimens that expressed α2 and α6 also did not have MYCN amplification, which is a characteristic of less aggressive tumors (29). To corroborate this, Judware and Culp found that decreased levels of α2 integrin was linked to overexpression of MYCN, suggesting that α2 could be protective against the oncogenic effects of MYCN amplification (31).

Favrot et al. also found that cells with higher expression of subunits α4, β4, β3, and αν were also non-MYCN amplified (29). This is currently up for debate, however, as another study by Gladson et al. suggested that higher expression of αν and β3 subunits, especially when combined to form ανβ3, could increase tumorigenic potential (32). Through hybridizing sections of neuroblastoma tumors at various stages, they saw that the β3 subunit was expressed at both the protein and mRNA levels in undifferentiated neuroblastomas, but it was not expressed in ganglioneuroblastoma (32). Undifferentiated tumors are more aggressive than ganglioneuroblastoma, so Gladson suggests that because this receptor is able to bind promiscuously to many different receptors, the upregulation of ανβ3 could lead to an increase in attachment, migration, and invasion (32). Meyer et al. also propose that ανβ3 integrin may enhance IGF-IR-mediated neuroblastoma cell migration and increase neuroblastoma cell attachment to ECM components (33). Favrot et al., on the other hand, found α4β1, ανβ3, and α6β4 were expressed in neuroblastoma tumors that developed in the mediastinum, a subset known to have a better prognosis compared to tumors that develop in the adrenal, and do not normally relapse (29, 34).

Two other integrins, α1β1 and α3β1, also have support for contributing to both favorable and unfavorable prognosis. Favrot et al. found that α1β1 and α3β1 were only observed in MYCN positive specimens, suggesting that they could be useful criteria for predicting a poor prognosis (29). Importantly, this β1 integrin/MYCN relationship is a potential target for stopping tumor progression. Sadasa et al. recently investigated a peptide derived from fibronectin, termed FNIII14, which inhibits β1 integrin (35). Since β1 integrin was overexpressed in MYCN amplified cells, using FNIII14 *in vivo* resulted in inhibited neuroblastoma tumor growth and consequently less MYCN amplification in the tumor cells (35). While it requires further study, their findings suggest that β1 integrin could be an important potential target for therapy. Villanueva et al. suggest that β1 integrin regulation could also be under the control of Neogenin-1 (NEO-1) (36). NEO-1 is a transmembrane receptor that associates with Focal Adhesion Kinase and goes on to activate β1 integrin. In their study, they found that NEO-1 did promote neuroblastoma migration and metastasis (36). However, some studies suggest that β1 expression is indicative of a better prognosis. Judware and Culp found that MYCN overexpression has an inverse relationship with reduced expression of α2, α3, and β1 (31). In MYCN amplified cells, there was also a two-fold decrease in β1 mRNA levels, suggesting that MYCN exerts a negative regulatory effect on transcription of β1. The authors note that an MYCN consensus binding site is in the β1 integrin promoter region, making this a plausible relationship (31). Meyer et al. concluded that primary neuroblastoma tumors with good prognosis express multiple β1 integrin heterodimers, while unfavorable neuroblastoma tumors lack β1 containing integrins (33). They also found that low-grade, differentiated neuroblastomas upregulate the expression of α1β1, α2β1, and α3β1 (33). These integrins are not found in higher-grade neuroblastoma specimens (37).

## Therapeutics Targeting CAMs in Neuroblastoma

Given that there is still a lot of controversy about which cell adhesion molecules are helpful versus harmful to neuroblastoma prognosis (**Table 1**) and given that CAMs are highly expressed on many normal tissues, it is not surprising that there are not many therapeutic options targeting CAMs currently. The two molecular targets that do seem promising for therapeutic intervention in neuroblastoma are N-cadherin and NCAM, which both have targeted therapies in the making.

**Table 1:** CAMs expressed in favorable neuroblastoma.

Cell Adhesion Molecule	Reference Number	Findings
T-cadherin	(13)	T-cadherin may function as a negative regulator of neural cell growth
N-cadherin	(9)	Downregulation of N-cadherin allow for migration and travel of tumor cells
	(12)	N-cadherin downregulation is associated with less cell-cell adhesion
NCAM	(16)	Cells with higher NCAM expression have more intense homophilic binding and less disaggregation from tumor sites
NCAM-120 isotope	(18)	More differentiated neuroblastic tumors have higher NCAM-120 expression
CHL1 and NrCAM	(20)	CHL1 and NrCAM are expressed in low-grade neuroblastoma tissue samples
ICAM-2	(28)	ICAM-2 is able to reverse the metastatic phenotype in neuroblastoma in the presence of α-actinin
α1 integrin	(30)	α1 expression is higher in non-MYCN amplified tumor cell lines
α2 integrin	(29)	Mainly expressed on low-grade, well-differentiated neuroblastoma specimens with no MYCN amplification
	(31)	α2 integrin is protective against the oncogenic effects of MYCN amplification
α4, αv, β3, β4 integrin	(29)	Higher expression found on non-MYCN amplified cells
α6 integrin	(29)	Mainly expressed on low-grade, well-differentiated neuroblastoma specimens with no MYCN amplification
α4β1, ανβ3, and α6β4 integrin	(29)	Expressed on mediastinal neuroblastoma tumors (better prognosis)
α3 integrin	(31)	Increased α3 integrin is associated with less MYCN amplification
β1 integrin	(31)	MYCN amplified cells have a decrease in β1 expression; MYCN has a negative regulatory effect on β1 transcription
	(33)	Primary neuroblastoma tumors with good prognosis express multiple β1 integrin heterodimers
α1β1, α2β1, and α3β1 integrin	(33)	Upregulation of these integrins is found in low-grade, differentiated neuroblastomas

N-cadherin, as mentioned before, is able to be targeted using alcohol dehydrogenase 1 (ADH-1), a cyclic pentapeptide that antagonizes N-cadherin and in turn causes rapid vascular disruption leading to apoptosis (38). In phase 1 trials, ADH-1 was shown to be well tolerated and demonstrated anti-tumor activity in patients with N-cadherin positive tumors (39). The patients tested included 46 subjects with incurable solid tumors (ovarian, breast, renal, head and neck) (38, 39). To date, there have not been any studies showing the efficacy of ADH-1 or ADH-1 conjugates in the treatment of neuroblastoma tumors, but this seems to be a promising target.

NCAM (CD56) has a therapeutic target currently under clinical trials. Lorvotuzumab mertansine (IMGN901) is an antibody-drug conjugate which specifically binds to CD56 and is also conjugated to DM1, a microtubule targeting agent (40). Neuroblastoma cells were sensitive to this treatment *in vitro* (40). IMGN901 went on through phase 1 and 2 trials, where it was tested in children diagnosed with Wilms tumor, rhabdomyosarcoma, neuroblastoma, synovial sarcoma, and pleuropulmonary blastoma (41). Throughout this trial, it was determined that IMGN901 was tolerated in children at the phase 2 dose, but there were only two out of 62 children in which a clinical response was noted (41). Out of those two patients, one was a patient with rhabdomyosarcoma who achieved a partial response, and the other was a patient with synovial sarcoma in whom a complete response was seen (41). Even though there was not a striking clinical usage for IMGN901, this model of an antibody-drug conjugate could be useful in future pharmaceutical development.

Markovsky et al. also performed a study on neuroblastoma cell cultures which targeted NCAM as a nanomedicine target (42). They made an NCAM-targeted polymer-drug conjugate that could be used to target tumors expressing NCAM. In their study, they describe the development and evaluation of an NCAM-targeted conjugate of polyglutamic acid with paclitaxel, which inhibited tumor growth (42). This provides the framework for another way that NCAM could be targeted to stop tumor progression.

## Conclusion

Despite many improvements to clinical care, neuroblastoma is still one of the hardest childhood cancers to treat, and current treatment options produce severe side effects (43). New therapies are still needed for children with advanced tumors that fail standard chemotherapy or surgical resection (44). Though CAMs are found in both healthy and tumor cells, the differential expression of CAMs in neuroblastoma cells versus healthy cells allows for it to be a possible therapeutic target (4). Targeting CAMs during tumor formation could slow progression to metastasis, as the cell adhesion molecules would not be able to either detach from the original tumor area or adhere to heterophilic cells (15). Cell adhesion molecules are an attractive molecular target, as their extracellular domains allow for antibodies or small-molecule inhibitors to bind easily to the target (1).

Though to this date there has not been any clinically significant therapies targeting cell adhesion molecules in neuroblastoma, there is a world of possibility for more effective pharmaceuticals to be uncovered (15, 40). Approaches that involve attachment to a cell adhesion molecule and subsequent conjugation with an apoptosis-inducing molecule are of particular interest (15). As there is still not a large pool of information surrounding the roles of cell adhesion molecules in neuroblastoma or a consensus on what overexpression is beneficial, future research will need to be done on the impact of cell adhesion molecules on neuroblastoma tumors (**Tables 1**, **2**). Future studies that include mechanisms of cell adhesion and metastasis are also paramount, as this will produce more effective and specific targeted therapies.

**Table 2:** CAMs expressed in unfavorable neuroblastoma.

Cell Adhesion Molecule	Reference Number	Findings
P-cadherin	(4)	Overexpressed in unfavorable neuroblastoma specimens
VE-cadherin	(10)	Stabilizes tumor vascular structures
OB-cadherin	(10)	Capable of conferring an invasive phenotype and may be involved in metastasis to bone tissue
NCAM-180 isotope	(18)	NCAM-180 is associated with cell motility and is highly expressed in undifferentiated neuroblastic tumors
PSA-NCAM	(17)	Decreases cell adhesion and increases cell migration
	(16)	PSA-NCAM allows for adhesion and penetration into endothelial cells
	(15)	PSA-NCAM complex keeps neuronal cells in an undifferentiated state
	(25)	Higher expression levels of PSA-NCAM are associated with higher-stage and more aggressive tumors
	(24)	PSA-NCAM is an even stronger unfavorable prognostic indicator than MYCN expression
ALCAM	(27)	Weak ALCAM expression is significantly correlated with established markers for poor prognosis, such as MYCN amplification and higher INSS stages
αvβ3 integrin	(32)	αvβ3 can bind promiscuously to many receptors, increasing attachment, migration, and invasion
	(33)	αvβ3 enhances IGF-IR-mediated neuroblastoma cell migration and facilitates attachment to ECM components
α1β1, α3β1 integrin	(29)	Only observed on MYCN positive specimens
β-integrin	(4)	Overexpressed in unfavorable neuroblastoma

## Author Contributions

BH was the primary author. CG conceptualized the manuscript and guided the content and edits throughout. Both authors approved the final version.

## Funding

This research was supported by the Jay A. Grosfeld, MD research grant from the American Pediatric Surgical Association (APSA) Foundation.

## Conflict of Interest

The authors declare that the research was conducted in the absence of any commercial or financial relationships that could be construed as a potential conflict of interest.

## Publisher’s Note

All claims expressed in this article are solely those of the authors and do not necessarily represent those of their affiliated organizations, or those of the publisher, the editors and the reviewers. Any product that may be evaluated in this article, or claim that may be made by its manufacturer, is not guaranteed or endorsed by the publisher.

## Abbreviations

CAMs, Cell adhesion molecules; MYCN, Myelocytomatosis viral related oncogene; NCAM, Neural cell adhesion molecule; PSA-NCAM, Polysialylated-neural cell adhesion molecule; ALCAM, Activated leukocyte cell adhesion molecule; ICAM-2, Intercellular adhesion molecule 2; (N-) cadherin, Neural; (E-) cadherin, Epithelial; (VE-) cadherin, Vascular endothelial; (OB-) cadherin, Osteoblast; DCC, Deleted in colon cancer; (P-) cadherin, Placental; EGF, Epidermal growth factor; (T-) cadherin, Truncated; CHL1, Close homolog of L1 protein; NrCAM, Neuronal glia related cell adhesion molecule; PSA, Polysialylated acid; NEO-1, Neogenin-1; ADH-1, Alcohol dehydrogenase 1; IMGN901, Lorvotuzumab mertansine.
